# Dynamic Self-Assembly of Polyelectrolyte Composite Nanomaterial Film

**DOI:** 10.3390/polym11081258

**Published:** 2019-07-30

**Authors:** Qiupeng Hou, Xiwen Wang, Arthur J. Ragauskas

**Affiliations:** 1 State Key Laboratory of Pulp and Paper Engineering, South China University of Technology, Guangzhou 510640, China; 2 Department of Chemical and Biomolecular Engineering, University of Tennessee, Knoxville, TN 37996, USA; 3 Department of Forestry, Wildlife and Fisheries, Center for Renewable Carbon University of Tennessee, Institute of Agriculture, Knoxville, TN 37996, USA; 4 Joint Institute for Biological Sciences, Biosciences Division, Oak Ridge National Laboratory (ORNL), Knoxville, TN 37831, USA

**Keywords:** dynamic self-assembly, polyelectrolyte multilayer films, silver nanoparticles

## Abstract

The aim of this study is not only to investigate the feasibility of using PAH (polyallylamine hydrochloride) and PSS (poly styrene-4-sulfonic acid sodium salt) to prepare a film via a layer by layer self-assembly process entrained with silver nanoparticles, but also to show that the silver nanoparticles crystalline structure can be defined and deposited on the surface of the substrate in the desired alignment structure and manner, which is of great help to research on the LBL method in the cellulose field. The effect of outermost layer variation, assembly layers, and composition of multilayers on the formation of the LBL structure on a nanofibrillated cellulose (NFC)/polyvinyl alcohol (PVA) substrate was investigated. The deposition of PAH and PSS was monitored by Fourier-transform infrared spectroscopy (FT-IR). The morphology of the LBL film layers was observed by scanning electron microscope (SEM) and atomic force microscope (AFM). Furthermore, thermal degradation properties were investigated by thermogravimetric analysis (TGA), and physical properties of multilayer films were tested by a universal mechanical tester. The results reveal that PAH and PSS can be readily deposited on a NFC/PVA substrate by using LBL methodology to prepare self-assembled polyelectrolyte multilayer films. The surface morphology of the LBL composite changed from negative to positive charged depending on the final LBL treatment. Also, according to SEM and AFM analysis, silver nanoparticles were well dispersed in the (PAH/PSS) film, which significantly improved the thermal stability of the composite films.

## 1. Introduction

At present, clean water resources are particularly important in our daily life. It was urgent to develop a pure water purification method, and thus membrane treatment technology came into being [1,2,3]. Furthermore, researchers have found that in the field of membrane applications and materials, composite materials have more potential development [4,5]. However, the biggest bottleneck is to continuously enhance the performance of composite membranes. For example, in the 1990s, researchers successfully applied LBL technology to form a film of 100 layers of alternating cationic and anionic polyelectrolytes on single-crystal silicon substrates [6]. This self-assembly methodology (LBL), which relies on the mechanism of strong intermolecular interactions (charge transfer interaction, electrostatic force, covalent bond interaction, hydrogen bonding, and so on) [7,8,9,10], has become one of the most frequently utilized processes for the preparation of functional multilayer films [11,12]. Moreover, adsorbed positively or negatively charged polyelectrolytic ions, can form polyelectrolyte–particle complexes by being exposed to light or chemical reagents. In recent years, nanomaterial films for water purification [13,14] have been prepared by LBL self-assembly method using polyelectrolyte polymers. The preparation process is an aqueous based process that yields an environmentally friendly synthesis protocol and provides well controlled membrane structures. Rubner and Cohen studied PEMs (proton-exchange membrane) produced from weak polyelectrolytes that were used to bind inorganic ions. The base substrate material (NFC/PVA) of these LBL studies combine the high elastic modulus advantage of NFC and the good solubility of PVA material [15,16]. The advantages of LBL technology have been discussed by many researchers, such as its ability to control the basic properties of the film, its unique characteristics, etc. The aim of this study is to investigate the feasibility of controlling the silver nanoparticles crystalline structure and its ability to deposit on the surface of the substrate in the desired alignment structure and manner, which is of significant use for research on the LBL method in the cellulose field.

Our research goal is to use LBL technology to produce (PAH/PSS: Ag) films. Dynamic LBL assembly technology utilizes the dilute solution containing organic or inorganic materials, flowing through the pore or surface of the porous support layer at a certain pressure, to form a separated layer of composite, thus preparing a composite membrane possessing special performance. It utilizes dynamic pressure to improve the compactness of the separation layer and it also use electrostatic interactions among the polyelectrolyte particles to overcome pure dynamic separation layer peeling defects to improve the quality of the surface. Recent studies have shown that nano Ag particles have promising biomedical and clinical applications, sensor and flexible electronics prospects and are being incorporated into a variety of materials [17,18,19]. PAH/PSS/nano-silver composite is a new composite which combines the beneficial properties of nano-silver and organic compounds, yielding a product with special attributes and applications, such as biocompatible ceramic materials, nanomembranes, and optoelectronic devices [20,21,22,23].

We will show that the silver nanoparticle crystalline structure can be defined. The effect of the outermost layer variation, the number of deposition bilayers, and the composition of the multilayers on the formation of the LBL structured NFC/PVA substrate was also investigated. Meanwhile, (PAH/PSS: Ag) film deposited on a stretchable NFC/PVA sheet under a controlled deformation procedure has attractive practical separation applications in the future.

## 2. Materials and Methods

### 2.1. Materials

The polyvinyl alcohol solution was prepared by dissolving polyvinyl alcohol particles (from Kuraray, Tokyo, Japan) with solid content of 0.5%, 87% hydrolysis rate (the hydrolysis rate represents the percentage of the hydrolyzed material to the total amount of substance, which is a basic property of the material), and viscosity of 40 Mpa. s into distillation (pH = 7). The natural wood fibers (bleached mechanical pulp, cork fibers, and Northern wood) used in the experiment were purchased from Guangzhou Paper Group Industrial Co., Ltd., Guangzhou, China. Pharmaceutical Group Industrial Corporation (Guangzhou,, China) provided analytical reagents, which were configured with deionized water as the solution used in experiment, such as 95% ethanol and 99.5% benzene (1:2 *v/v*), diluted hydrochloric acid (1.19 g/mL), 99.7% sodium chlorite (NaClO_2_), 85% potassium hydroxide (KOH) solution (6 wt.%), 99% sodium bromide, 99% tetramethylpiperidine nitrogen oxides (TEMPO), 99.7% sodium hydroxide (NaOH), AgNO_3_ solution (15 mL 9 mg/mL), and 96% NaBH_4_ (0.01M). Poly (allylamine hydrochloride) (PAH, Mw = 1.5 × 10^4^ g/mol) and poly (styrene-4-sulfonic acid sodium salt) (PSS, Mw = 7 × 10^4^ g/mol) were purchased from Aladdin chemical Ltd, Shanghai, China. All solutions were prepared at pH = 5 and at standard room (25 °C); the concentration of silver nitrate solution is 10 mg/mL^−1^, prepared with distilled water.

### 2.2. NFC Generation

In the experiment, the Soxhlet extraction method was used to separate the extract from the bleached softwood mechanical pulp by using a standard solution (ethanol and benzene (1:2 *v/v*)) and the entire process was continued for 48 h. Next, the lignin components were removed. Configured acidic solution (pH = 4–5) was placed at a temperature of 70 °C, then it was repeatedly soaked in a sodium chlorite (NaClO_2_) solution 4 times, and finally washed with deionized water for 1 h. Finally, the cellulose was immersed in a potassium hydroxide (KOH) solution (6 wt.%) at a temperature of 20 °C for 24 h, in order to remove hemicellulose [24].

Under the room temperature and stirring conditions, absolutely dry cellulose fibers (2 g) were poised in a 150 mL mixed solution (0.250 g sodium bromide: 0.025 g TEMPO). With the continuous dripping of 13% NaClO_2_ solution, a TEMPO oxidized cellulose slurry was made. Then, an NaOH solution (0.5 M) was slowly added to the aforementioned solution to bring the pH value to 10.5 without further reduction, indicating that the oxidation reaction was completed. Next, the pH value of the suspension was adjusted to 7 by adding HCl solution (0.5 M) [25]. Finally, oxidized cellulose was thoroughly washed with water and stored at 4 °C for subsequent treatment or analysis.

### 2.3. Preparation of NFC/PVA Substrate

A nanofibrillar cellulose solution (0.5 mg cellulose/L) was dispersed with a 0.5 wt.% PVA solution and stirred for 2 h (600 Hz) at 25 °C. The slurry was filtered onto a microfiltration membrane following a papermaking process [26,27] and after drying a NFC/PVA film was obtained that was 80 μm thick.

### 2.4. Preparation of PAH and PSS Film

PAH and PSS were alternatively deposited on a film of nanofibrilled cellulose/PVA planar substrates obtained from above NFC/PVA substrate discussion by means of LBL technique. Each NFC/PVA substrate was alternatively dipped in 2 mg/mL PAH or PSS or PSS-Ag solutions, and then, using water to rinse, intermittent dispersion lasted for 5 min. The PSS-Ag solution was prepared by adding the PSS solution (30 mL 2 mg/mL) drop-wise in the AgNO_3_ solution (15 mL 9 mg/mL). After that, the LBL assembly materials (PAH/PSS/PAH/PSS-Ag films) were impregnated in the 0.01 M NaBH_4_ dispersions to stabilize the reaction of oxidized cellulose for 15 min and then washed and dried.

## 3. Results and Discussion

### 3.1. Surface Charge

**Figure 1:**
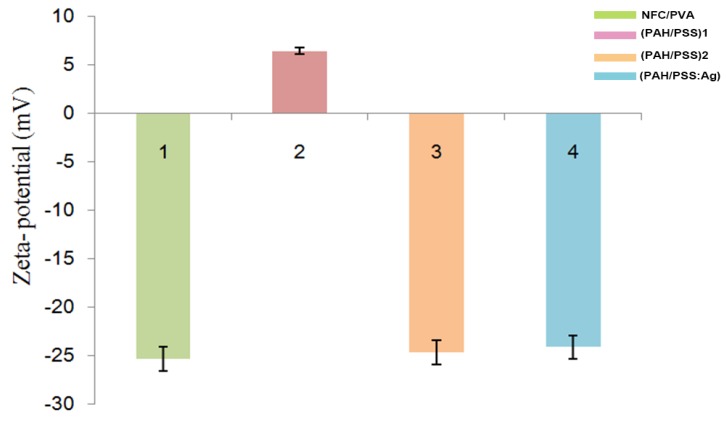
Zeta potential of the surface charge of (PAH/PSS) multilayer film, including (NFC/PVA) substrate (1), (PAH/PSS) _1_ (2) means only PAH was deposited on NFC/PVA substrate, (PAH/PSS) _2_ (3) means PSS was deposited on NFC/PVA substrate surface, (PAH/PSS: Ag) (4) means a total of 2 bilayer films composite of PAH or PSS material; PAH and PSS were alternatively deposited on NFC/PVA substrate and Ag was deposited on the top surface of the film.

The electrophoretic mobility is measured by using a motorized analyzer (SurPASS, Anton Paar GmbH, Graz, Austria) which has a flat solid sample measurement unit. In addition, each sample needs to be soaked in 10^−3^ mol/L KCl solution for 24 h before measurement. Only when the sample is thoroughly cleaned, it could be measured [28,29]. During the self-assembly of PAH /PSS layers, we monitored the zeta potential to characterize surface charge as summarized in Figure 1. The results show that the surface potentials of membranes with different polymers (PAH or PSS) exhibited varied Zeta potential under neutral conditions.

As shown in Figure 1, due to the presence of PAH, zeta potential measurements showed that this (PAH/PSS) _1_ surface has a positive charge (6.39 ± 2.4 mV, pH = 7). When a layer of PSS was assembled on the PAH membrane, the surface potential reversed to negative values, indicating that PSS assembled on the surface of the PAH films. However, after assembling a layer of PSS, the surface potential of the films was below −24 mV [30,31,32]. Meanwhile, after the LBL assembly of PSS-Ag, there is a strong interaction between PSS, PAH, and Ag+ resulting in the partial ionization of amino groups on PAH [33,34,35]. Thus, in the process of assembling the LBL films, the surface charge of the composite films changed alternately with the change of the outer polyelectrolyte, and the positive and negative potentials changed alternately.

### 3.2. Fourier-Transform Infrared Spectrometry (FT-IR)

**Figure 2:**
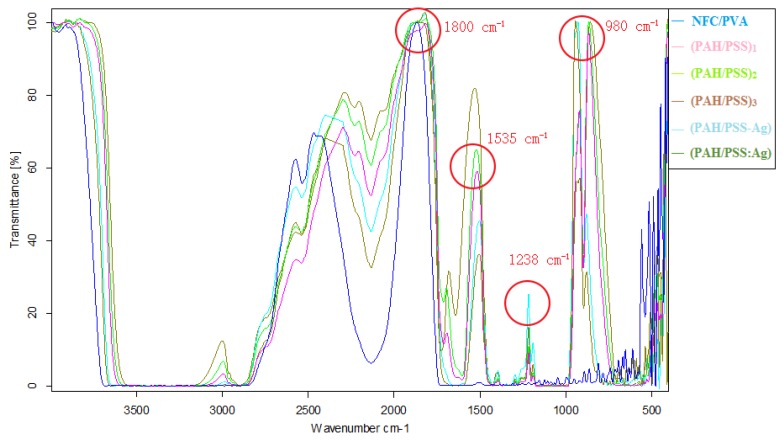
FTIR spectra of (NFC/PVA), (PAH/PSS) _1_, (PAH/PSS) _2_, (PAH/PSS) _3_, (PAH/PSS–Ag+), and (PAH/PSS: Ag) (**a**–**f**).

In transmission mode, the results of FT-IR spectral irradiation (VERTEX 70, Fourier Transform Infrared Spectrometer, Bruker Optics, Inc., Karlsruhe, Germany) were recorded at the condition of a resolution of 4 cm^−1^. The resulting transmittance is converted to the indicated absorbance unit. FT-IR analysis was used in this study to verify the presence of PAH and PSS [36,37,38]. The infrared spectra of different samples are shown in Figure 2.

As shown in Figure 2, the main absorption peaks of NFC/PVA LBL substrates are as follows: in the vicinity of 1238 cm^−1^, the asymmetric tensile vibration peaks of the base bond and the C-O-C bond of the cellulose pyran ring appeared, respectively; the tensile vibration peak of C-O appears at 980 cm^−1^, the common band of carboxyl group was observed at 1535 cm^−1^, indicating this LBL method was effective.

In addition, the skeleton vibration peak of an aromatic ring observed at 1600 cm^−1^ and the carbon-nitrogen bond occurred at 800–920 cm^−1^, which all signify the presence of PAH [39]. Furthermore, the NFC/PVA film has no absorption peaks in the vicinity of 1190 cm^−1^ but a broad signal appeared in the LBL films, which is characteristic of the sulfate group of PSS [40], and its absorption intensity increases with the increasing number of assembly layers. This showed that the amount of assembly of PSS increased with the number of assembly layers. At the same time, the amount of PSS adsorbed on each newly assembled layer is very close. Moreover, the carbonyl groups (wavenumber 1650–1800 cm^−1^) have the strongest absorption peak, and it is also suggestive of O–NO_2_ which appears in the vicinity of 1630–1275 cm^−1^ [41].

### 3.3. Morphology Observation

SEM (Scan Electron Microscope, Oxford Instruments, Oxford, UK) was operated at an operation voltage of 10 kV. All the samples used an SPI sputter coater to enhance the conductivity (2–4 nm gold layers). AFM (Atomic Force Microscope, Bruker Optics, Inc., Karlsruhe, Germany) was also used to analyze morphology of the cellulose substrate and (PAH/PSS) multilayer film (tapping mode).

**Figure 3:**
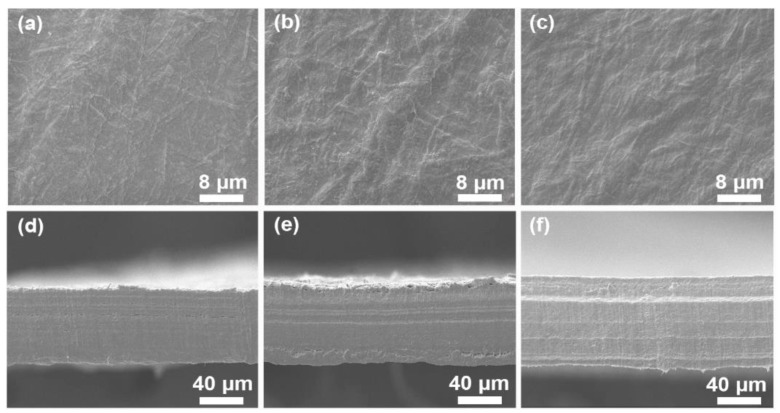
SEM of surface morphology of PAH and PSS film deposited on NFC/PVA substrate (**a**), (NFC/PVA) (**b**), (PAH/PSS) _1_ (**c**), (PAH/PSS) _2_ and cross-section of (NFC/PVA) substrate (**d**), cross-section of (PAH/PSS) _1_ polymer film (**e**), and cross-section of (PAH/PSS) _2_ polymer film (**f**).

As shown in Figure 3, the surface of the NFC/PVA substrate is smooth. Furthermore, the outer layer of (PAH/PSS) _1_ film is a PAH layer and its surface is complete and compact, and there are many different sizes of convex (the diameter of the bulge is approximately 70 to 180 nm). This kind of protrusion may be caused by the aggregation of PAH molecules, because of the well compliant PAH molecular chain whose conformation is easily formed in the solution. On the other hand, the increase in density and the presence of small protrusions were attributed to the hydrolysis of PAH [42,43,44]. The increase of density is beneficial to the bridging connection of the polyelectrolyte on the surface of the substrate membrane pore. Moreover, the protrusions can increase the contact area of the polyelectrolyte and the supporting substrate membrane. The negative charge of the membrane surface increased and it is beneficial to the subsequent ion adsorption, enhancing the binding of assembled layer and basement membrane.

The initial PSS film on NFC/PVA is similar to (PAH/PSS) _1_ morphology, and there are also different sizes of projections, but the number of protuberances is less and relatively small (the diameter of the bulge is ~30–100 nm). This is because after the PSS layer covers the surface of (PAH/PSS) _1_ film, the small protrusions on the surface of the original (PAH/PSS) _1_ film are completely covered, while the larger ones are partially covered. These results suggest that there are interlayer PAH aggregates on the surface of the (PAH/PSS) _1_ membrane.

**Figure 4:**
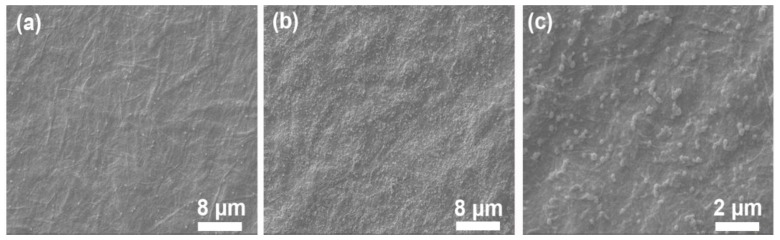
SEM of surface morphology of (PAH/PSS) film (**a**), (PAH/PSS-Ag) _2_ (**b**), and (PAH/PSS: Ag) _2_(**c**).

From the point of view of the generated silver nanoparticles, scanning electron microscopy (SEM) observation shows from Figure 4 that the surface of PSS membrane is flat and smooth, and there are many different sizes of convex. This kind of protrusion is due to the stress action of the thin film in the production process. The stress concentration in the weak part of the film produces local plastic deformation and orientation in the thin film. At the same magnification, Figure 4b illustrates a larger volume of silver nanoparticles and silver particle aggregation. Thin film silver particles can be seen in Figure 4c, without aggregation of silver nanoparticles, presenting a uniform distribution of (PAH/PSS) composite film.

**Figure 5:**
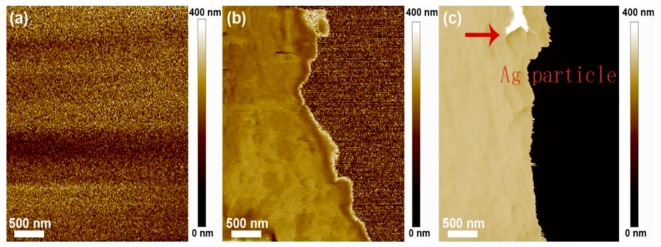
Atomic force microscopy (AFM) of surface morphology of a LBL polymer film NFC/PVA substrate (**a**), (PAH/PSS) _1_ film (**b**), and (PAH/PSS-Ag) _2_ nanomaterial film (**c**).

As shown in Figure 5b, the image with PAH surface layer composition was smoother than that of Figure 5a (NFC/PVA substrate film) due to the penetration of PAH into the voids of the substrate. In addition, the boundary exists in Figure 5b, because this is the intermediate state (partial covering) of the LBL assembly layer, that is, PAH is covering the surface of the substrate through the LBL self-assembly process. Furthermore, as shown in Figure 5c, images with PSS surface layer components were also smoother than NFC/PVA basement membranes [45,46,47]. It was observed that the film surface became more uniform and smooth as the number of layers increased [48].

### 3.4. Thermal and Physical Properties

In experiment, the thermal shrinkage of the substrates was measured by thermogravimetric method (TG) (Q50 Thermogravimetric Analyzer, TA Instruments, Newcastle, DE, USA) and the average pore size of the substrates was measured with a capillary flow meter (PMI Capillary Flow Porometer, Porous Materials Inc., NY, USA).

The pore size of NFC/PVA substrate decreased with the deposits of PAH and PSS, and the surface structure of the film became more compact, as shown in Table 1. In addition, the pore size decreased with the increase of the number of assembly layers, i.e., polymer content. Also, the multilayer films prepared by the LBL method exhibited excellent tensile strength and modulus of elasticity due to its high electrostatic interaction in molecular domain [49].

**Figure 6:**
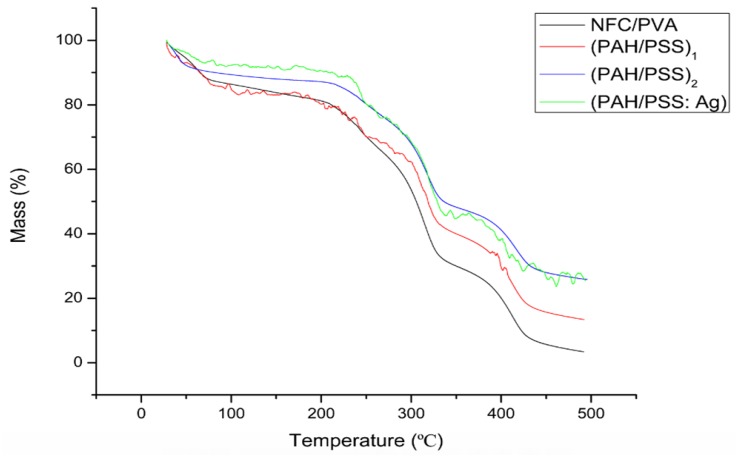
TGA thermograms of NFC/PVA substrate, LBL structure NFC/PVA substrate coated with (PAH/PSS) _1_, (PAH/PSS) _2_, and (PAH/PSS: Ag).

As shown in Figure 6, the first stage caused by the loss of bound water in films occurred around 59 °C, and the mass loss of membrane was ~7%. The second stage caused by the thermal degradation of PVA occurred within 210–450 °C and it mass loss was 72%. It should be noted that the degradation temperature of pure NFC/PVA film was significantly lower than that of composite film due to the strong electrostatic interaction between PAH and PSS molecules, which increased the heat resistance of multilayers. After 500 °C, the carbon-containing materials began to decompose, that is, the third weightlessness stage of 480 °C. Furthermore, the increase of thermal decomposition temperature of (PAH/PSS: Ag) also suggests that the addition of Ag nanoparticles can aid in improving the thermal stability of the (PAH/PSS) film.

## 4. Conclusions

The (PSS/PAH) polyelectrolyte composite membrane was prepared on the porous NFC/PVA substrate film by using LBL electrostatic assembly technology. FT-IR, AFM, and SEM studies showed that PAH and PSS through electrostatic interaction can self-assemble polyelectrolyte multilayer films. When the NFC/PVA substrate assembled polyelectrolyte multilayer film, its surface morphology changed from neutral to positively or negatively charged, and thus has a certain ion selective retention. In addition, we have successfully assembled horizontally oriented Ag nanoparticles by decreasing of PSS-Ag in LBL technology. At the same time, the (PAH/PSS: Ag) composite film was effectively prepared by NaBH_4_. Moreover, the silver nanoparticles in (PAH/PSS: Ag) composite films were dispersed well in (PAH/PSS) without obvious aggregation of silver particles, which also improved the thermal stability of the composite films by 15%.

## Figures and Tables

**Table 1:** Physical properties of (PAH/ PSS) multilayer film.

Multilayer Film	Mean Pore Size (μm)	Elastic Modulus (GPa)	Tensile Strength (MPa)
NFC/PVA	7.53	0.25	12.94
(PAH/PSS) _1_	6.75	0.83	6.82
(PAH/PSS) _2_	5.54	4.29	21.90
(PAH/PSS: Ag) _2_	0.28	3.62	28.17
